# Supplementary material for: Evolution of phenotypic fluctuation under host-parasite interactions
Source: PLoS Comput Biol. 2021 Nov 9;17(11):e1008694. doi: 10.1371/journal.pcbi.1008694 (PMC8604345; doi:10.1371/journal.pcbi.1008694)
Supplement: S1 Appendix — Fig A: Evolutionary change of the variances Vip′t for output genes for c = 0. Fig B: Average variances of Vip(i) and Vg(i) (non-target output genes i = 4, 5, …, 8). Fig C: Average variances of Vipt and Vgt (target genes i = 1, 2, 3). Fig D: Average variances of Vip(i) and Vg(i) (other genes i = 9, 10, …, 64). Fig E: Evolutionary time course of the average phenotypic variations Vip and Vip′. Fig F: Evolutionary change of the variances for target genes and output genes for c = 0 and c = 3. Fig G: Time course of the variances Vipt and the growth rate μ and μ^. Fig H: Timescale of the parasite changes. Fig I: Phenotypic variance Vip and Vg of the output genes against the parasite interaction strength. (PDF) [file pcbi.1008694.s001.pdf]

Supplementary Information of
Evolution of Phenotypic Fluctuation under Host–Parasite Interactions

Naoto Nishiura[1], Kunihiko Kaneko[1,2*],

1 Department of Basic Science, Graduate School of Arts and Sciences, University of Tokyo, Tokyo, Japan **2** Center for Complex Systems Biology, Universal Biology Institute, University of Tokyo, Tokyo, Japan

A Supplement

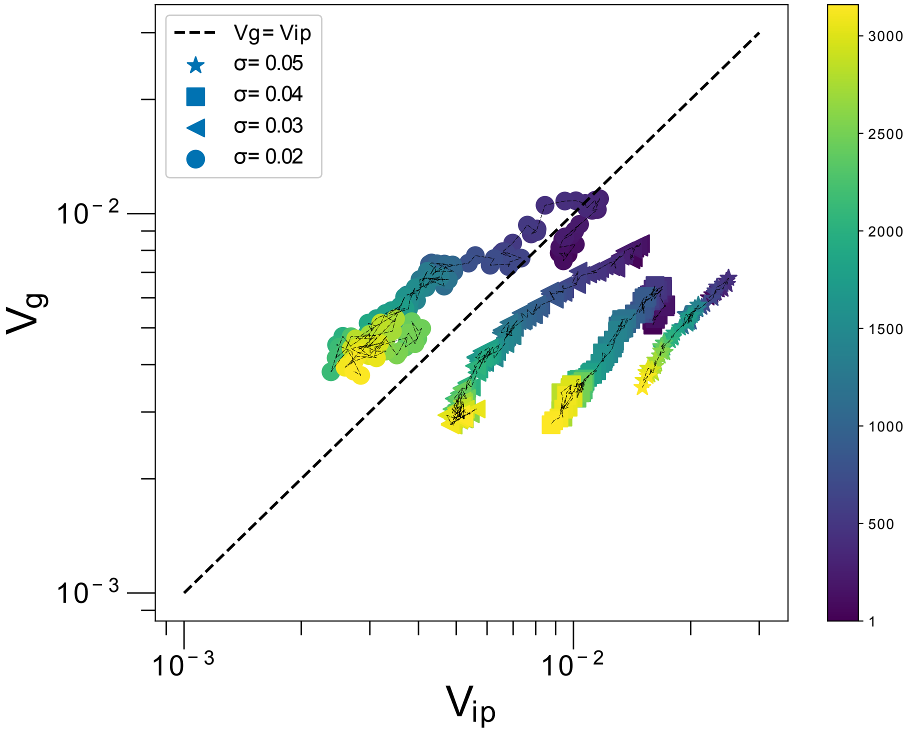

Fig A. Evolutionary change of the variances for output genes for $c = 0$. The time course of the variance (V_{ip}, V_g) for output genes ($\sigma = 0.05$ ($\star$), $\sigma = 0.04$ ($\diamond$), $\sigma = 0.03$ ($\triangleleft$) and $\sigma = 0.02$ ($\circ$)). The variances are computed from the isogenic variance over 100 iterations. The time course over generations is plotted for the variances of gene expression. The plots cover 3000 generations. At noise strengths above $\sigma_c = 0.02$, the two variances decrease throughout the evolutionary course.

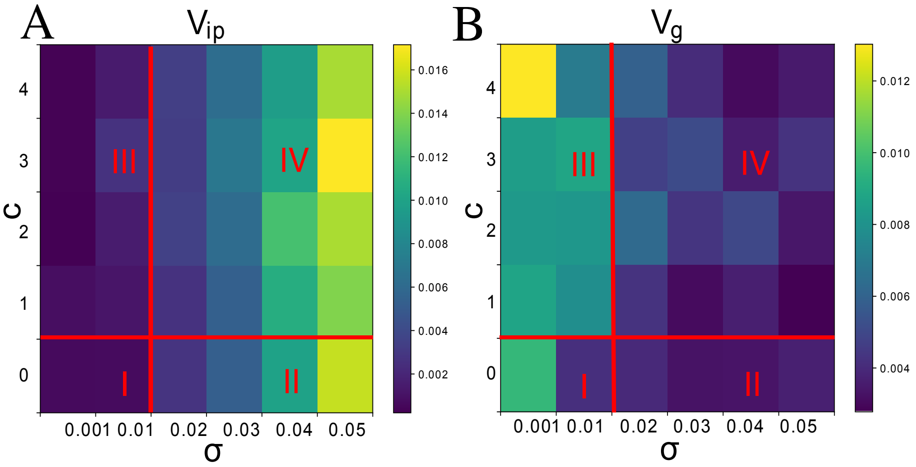

Fig B. Average variances of $V_{ip}(i)$ and $V_g(i)$ (nontarget output genes $i = 4, 5, ..., 8$). Dependence of $V_{ip}(i)$ and $V_g(i)$ for the nontarget output genes $i = 4, 5, ..., 8$ on the noise level σ (horizontal axis) and the interaction strength c (vertical axis). The variance values for each parameter are displayed as color maps according to the color bars. The variance is computed by averaging across 2500–3000 generations.

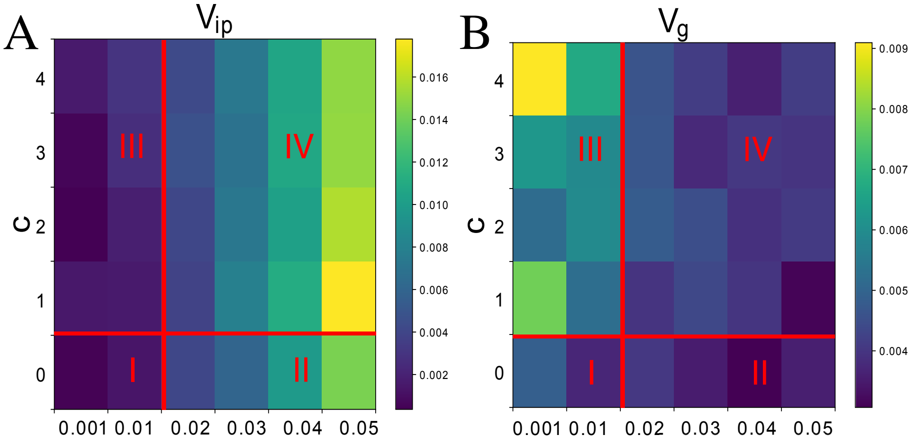

Fig C. Average variances of V_{ip}^t and V_g^t (target genes $i = 1, 2, 3$). Dependence of V_{ip}^t and V_g^t for the target genes on the noise level σ (horizontal axis) and the interaction strength c (vertical axis). The variance values for each parameter are displayed as color maps according to the color bars. The variance is computed by averaging across 2500–3000 generations.

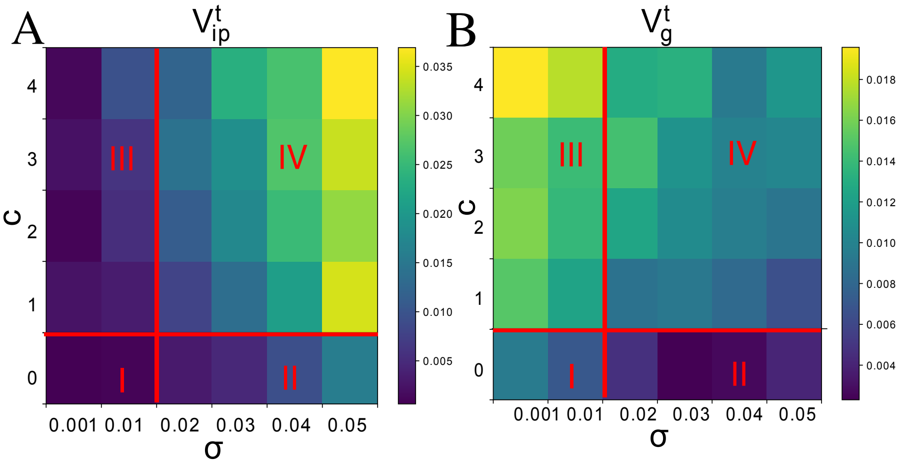

Fig D. Average variances of $V_{ip}(i)$ and $V_g(i)$ (other genes $i = 9, 10, ..., 64$). Dependence of $V_{ip}(i)$ and $V_g(i)$ for the non-output genes $i = 9, 10, ..., 64$ on the noise level σ (horizontal axis) and the interaction strength c (vertical axis). The variance values for each parameter are displayed as color maps according to the color bars. The variance is computed by averaging across 2500–3000 generations.

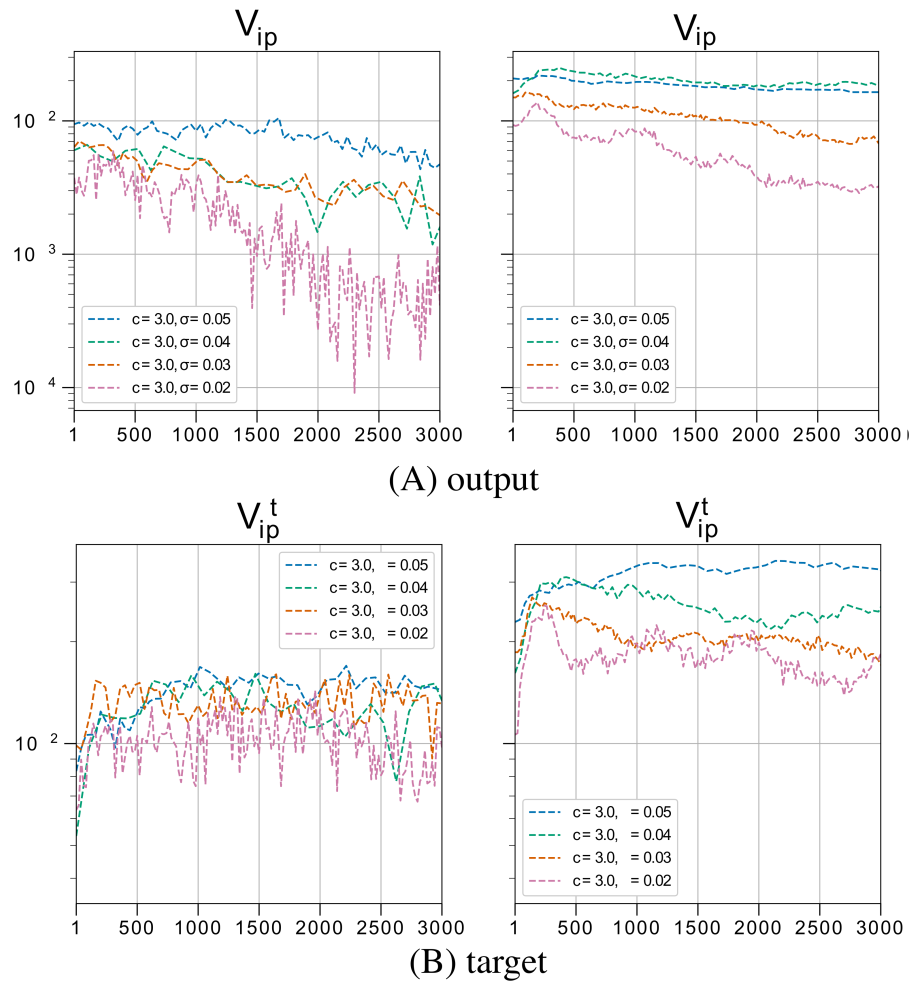

Fig E. Evolutionary time course of the average phenotypic variations V_{ip} and $V_{ip}{}'$ for $c = 3$. The time course of the average phenotypic variations V_{ip} and $V_{ip}{}'$ (i.e., V_{ip}^t and $V_{ip}^{t}{}'$). (A) Average V_{ip} and $V_{ip}{}'$ over all output genes are plotted against the generation for different values of the noise level σ, as indicated by different colors. The time course of the variance (V_{ip} and $V_{ip}{}'$) for output genes ($\sigma = 0.05$ (blue), $\sigma = 0.04$ (green), $\sigma = 0.03$ (orange) and $\sigma = 0.02$ (red)). Both V_{ip} due to white-noise fluctuations and $V_{ip}{}'$ due to noise-induced switches decrease compared to the initial network, and the developmental robustness evolves. (B) V_{ip}^t and $V_{ip}^{t}{}'$ for the target genes. The line colors represent the equivalent conditions as in (A). Both V_{ip}^t and $V_{ip}^{t}{}'$ increase from its initial level through the evolution and maintains a high value. We used the function $z_i = tanh\beta(x_i - 0.5)$ with $\beta = 100$ and defined $V_{ip}'(i)$ as the variance of z_i.

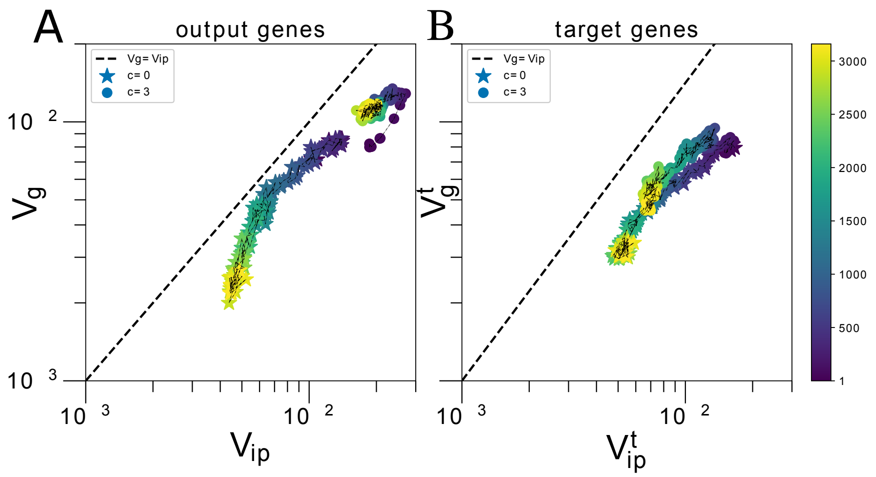

Fig F. Evolutionary change of the variances for target genes and output genes for $c = 0$. The time course of the variance (V_{ip}^t, V_g^t) for target genes (A) and the variance (V_{ip}, V_g) for output genes (B) ($c = 0$ (asterisks) and 3 (circles)). The variances are computed from the isogenic variance over 100 iterations. The time course over generations is plotted for the variances of gene expression. The plots cover 3000 generations. We set the noise level to $\sigma = 0.03 > \sigma_c$. In the absence of parasite interactions ($c = 0$), the two variances decrease, while $V_g < V_{ip}$ is maintained throughout the evolutionary course. Conversely, under host-parasite interactions, V_{ip} and V_g show a correlated increase. Although this increase is prominent for target genes (asterisks in (A)), it is suppressed for output genes, before showing a slight decrease (B).

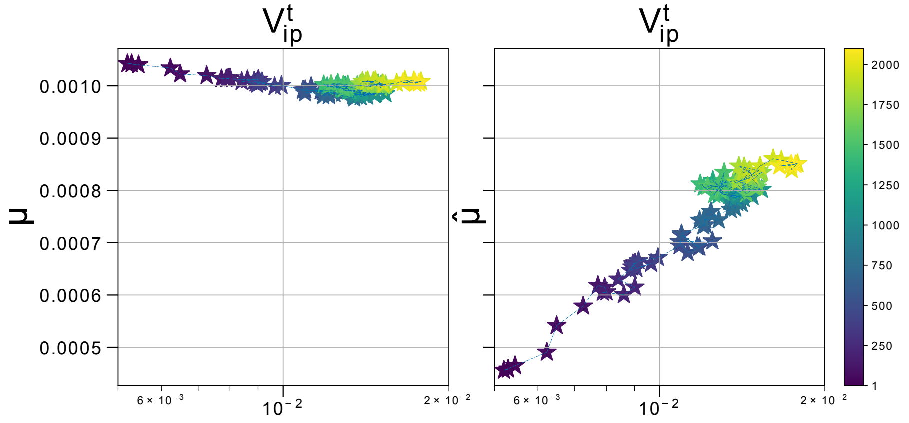

Fig G. Time course of the variances V_{ip}^t and the growth rate μ and $\hat{\mu}$. The time course of the variances (V_{ip}^t, μ) and (V_{ip}^t, $\hat{\mu}$) over generations for $\sigma = 0.03$. For the first 2500 generations, the evolution of the host was modeled without parasites ($c = 0$), after which the evolution of robustness was complete. Then, we introduced the interaction with the parasites by changing c from 0 to 3. V_{ip}^t is computed from the isogenic variance over 20 iterations. The subsequent time course of (V_{ip}^t, $\hat{\mu}$) and (V_{ip}^t, $\hat{\mu}$) over generation is plotted for the target genes $i = 1, 2, 3$. The host growth rate $\hat{\mu}$ increases with the evolution of plasticity (V_{ip}^t). Other model parameters: $N = 300, M = 64, l_{inp} = 8, l_{out} = 8$, and $l_p = 3$.

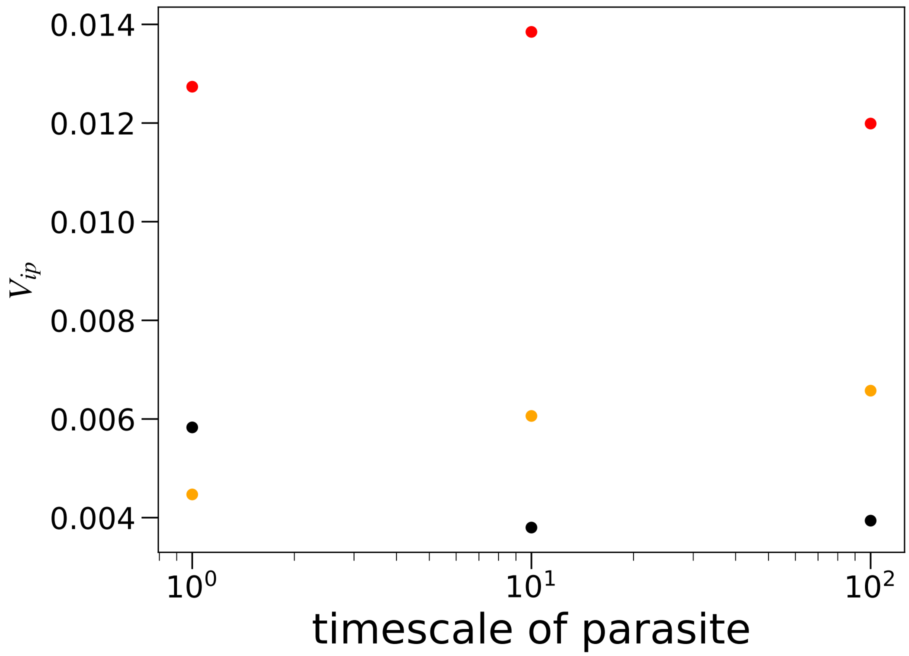

Fig H. Timescale of the parasite changes. Relation between the phenotypic fluctuations (V_{ip}^t) of the target genes and the timescale of parasite dynamics T_p, computed by averaging across 2500–3000 generations. Each color represents the noise strength $\sigma = 0.01$(black), $\sigma = 0.015$(yellow), $\sigma = 0.03$(red)

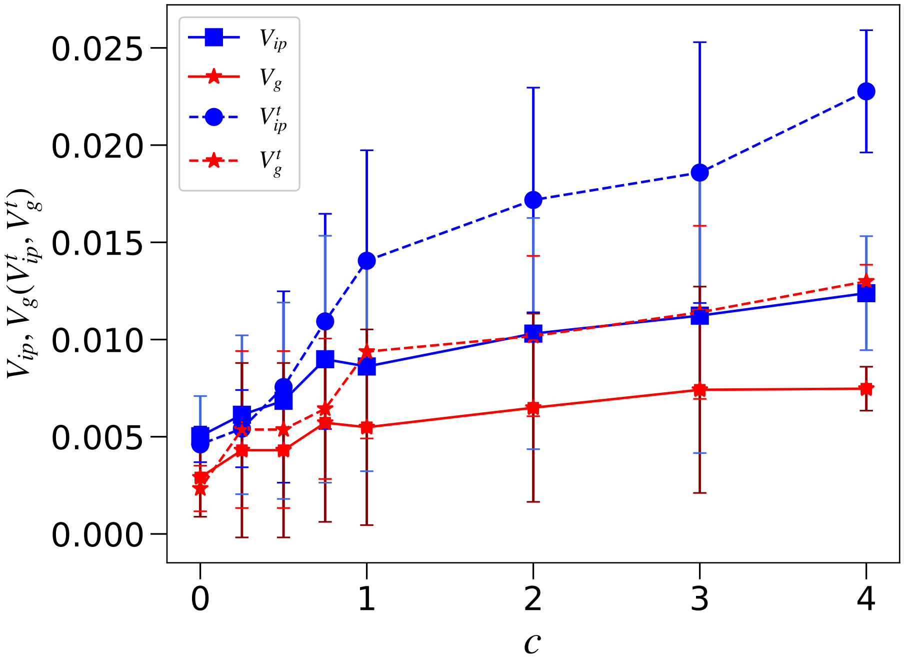

Fig I. Phenotypic variance V_{ip} and V_g of the output genes against the parasite interaction strength. Dependence of the average variances V_{ip} (red) and V_g (blue) for output genes (solid lines) and V_{ip}^t (red) and V_g^t (blue) for target genes (dotted lines) upon the interaction strength c for a strong noise level ($\sigma = 0.03$). V_{ip} and V_g are computed from the expression levels of the output genes over the host population ($N = 300$ individuals) and plotted as the average V_{ip} (V_g) over 2500–3000 generations.
