# Supplementary material for: Evolution of phenotypic fluctuation under host-parasite interactions
Source: PLoS Comput Biol. 2021 Nov 9;17(11):e1008694. doi: 10.1371/journal.pcbi.1008694 (PMC8604345; doi:10.1371/journal.pcbi.1008694)
Supplement: Attachment — Submitted filename: Review-PCOMPBIOL-D-21-00026_Kaneko.pdf [file pcbi.1008694.s002.pdf]

Report on the article: "Evolution of Phenotypic Plasticity under Host-Parasite Interactions" by Naoto Nishiura and Kunihiko Kaneko

In their work Nishiura and Kaneko investigate the behaviour of a model that represents evolution of an organism with dynamical genotype-phenotype correspondence in an environment with a simple ecological feedback. The subject of the work is crucial for an extension of the current main stream evolutionary theory. A need for such an extension is being gradually recognised by more and more researches in the field in recent years. The two main directions of the extension are 1) a nontrivial dynamic mapping from genotypes to phenotypes typical for organisms with complex development and 2) an embedding of an evolving system into an ambient ecosystem of other evolving systems. The current work attacks both directions using a numerical model. The model formalises the direction 1) by sandwiching a dynamical system between a genotype and its phenotypic manifestation, where the former provides parameters to the dynamical systems while the latter is understood as a certain characteristics of its stable regimes. The dynamical systems is a version of a simple model for a noisy gene regulation network (GRN). This part of the work is not new *per ce* and utilises an earlier published model of one of the authors (I will cite only [1], but there is a whole series of works on the topic). The direction 2) is implemented *via* a simple host-parasite co-evolution scheme. The hosts are modelled with the aforementioned noisy dynamical genotype-phenotype correspondence while the parasites, which constitute a dynamic background for evolution of the hosts, a modelled in a more simplified way. The evolution of hosts is modelled by numerical simulation of a stochastic evolving system with a fixed population size akin of the Moran process. The parasites follow a simple infinite population diffusive approximation of the Write-Fisher process with a simple functional genotype-fitness mapping. All conclusions are obtained by simulations and numerical solutions of differential equations.

The main problem studied in the work is the investigation of a possibility to evolve phenotypic plasticity due to environmental fluctuations in form of the host-parasite dynamics. It was shown in [1] that under the conditions of the fixed fitness landscape on the phenotypical space a system with a dynamical genotype-phenotype correspondence (the same as in the current study) evolves towards increased robustness both to the internal dynamical noise and to mutations, which in turn decreases phenotypic variability of the resulting population. The latter is seen by the authors as an obstacle for evolvability of the system. In the current work the authors investigate if a co-evolutionary

coupling with the environment can force the system to maintain phenotypic variability despite the earlier demonstrated, and much earlier theoretically expected, tendency towards canalisation.

The main conclusions of the work are the following. In the space parametrised by c (the strength of parasitism) and σ (the noise strength of the developmental process) there are four different phases. The two phases with c small repeat the two phases observed in [1]. The two newly observed phases correspond to the different response strategies to the parasite load (high c): when σ is small a genetic variability of the population is increased, while when σ is large, the phenotypic variability is increased by the decrease of the robustness against the noise. Interestingly, while this decrease is the strongest for the phenotypic traits (metabolites) that are in direct interaction with parasites, a lesser degree of decanalisation is observed in all other traits, too.

Although the ideas on the consequences of a dynamic relation between genotype and phenotype (epigenesis *sensu* Waddington) and its relation to canalisation date back to the first half of the XX century (Schmalhausen, Waddington), up to this day, with an exceptional attempt by R. Thom in the 60-80s, these ideas were not properly formalised. The current manuscript is one of the series of steps in this direction undertaken by the authors in an original way. Although the basic elements (the model of GRN, the host-parasite co-evolution formalism) are not original, bringing them into the context of evolution of an organism with development in an ecosystem is.

The novelty of the current work with respect to the earlier works (like [1]) is the inclusion of the ecological dynamics mediated by the co-evolving parasites. The novelty of the series of the work by Kaneko to which this manuscripts belongs is in a formalisation of development *via* a specific finite-dimensional dynamical system related to models of GRNs (a realisation of the idea by Delbrück from the beginning of the XX century).

The importance of the work to the development of an adequate theory of evolution is difficult to overestimate, but this already must by clear from the introductory paragraph of this review.

The scientific quality and validity of the work is on the high level.

I would recommend the article for publication in PLOS Computational Biology as is (and I think it is very important to publish this work in general) if not for some incompleteness of the Methods description, which constitutes my main critique. There are also a number of lesser faults elimination of which would benefit the article. I elaborate them below.

Here is the list of main issues that are to be addressed:

My main concern is the incompleteness of the methods in section *Models* and the *Methods* section itself. It is impossible to repeat the numerical experiments based on the description given in them. The main problems are the following.

1) The *Fitness and reproduction* part describes mutations during the reproduction (line 132), but it is not written how often they were added to J_{ij}. There is no clear parameter that controls the mutation rate in Table 1. Instead, there is a parameter T_p named "evolutionary speed" with no explanation of what it is. Perhaps it is somehow related to the mutation rate but this stays obscure. The same problem

exists for the parameter D_p, the mutation rate of the parasites. It is neither listed in Table 1 nor its value is revealed anywhere in the text.

2) It is not clear how exactly to compute $V_{\text{ip}}^{(l)}$. First, the term "host-type" (line 388) should not be used for the l-th organism in the population, as it brings confusion (see below in the minor comments). Second, when $V_{\text{ip}}^{(l)}$ is computed is it that only (1) is numerically solved for the same J_{ij} but different initial x and noise realisations, or is it that (1) is solved along with the simulation of the population growth by (2), but with suppressed mutations and no parasite attack, or something else? It is not clear from the description. The two situations differ in the presence or absence of the random re-initialisation of the dynamics at a random initial datum in (1) after each division event in (2), which can lead to a situation when at large time the population has a substantial fraction of unsettled in terms of their internal metabolic state individuals. Likewise it is not clear how to compute $V_{\text{g}}^{(l)}$: with respect to only metabolic simulation (1) or the full population simulation (1)–(2).

3) There is a contradiction in the value of the external input I_j. On line 103 it is indicated that $I_j = 1$. In Table 1 it is indicated that $I_j = -5$. Obviously, from these two only 1 can represent the real value. What was used in the actual numerical experiment?

4) How are the parasites P_i initialised?

Here are some minor comments and typos:

1) There must be a typo in the indices of J-s at line 135 describing the mutation process that conserves α. Right now the description is nonsensical.

2) Line 92. θ_i is not simply randomly selected but from a uniform distribution, isn't it? The same goes for x_i during the re-initialisation after a division.

3) Line 93. Please, elaborate why $\sqrt{\alpha M}$ is the correct scaling factor. It is not obvious for me.

4) In my taste exp, l_{inp}, l_{out} better look as $\exp$, l_{inp}, l_{out}. Of course, this is a matter of taste, not a requirement.

5) Line 109. What does "ds" mean? Must be a typo.

6) Line 143 and line 159. Almost the same notation (v_i and υ_i) is used for two different entities. Furthermore, $\upsilon_i^{(l)}$ introduced on line 159 (the variance of the gene expression of $x_i^{(l)}$) is never used in the text after that place. Was it a typo?

7) Fig. 8. On this figure and in its caption the quantity $\sum_i^{l_{\text{out}}} x_i$ is called *fitness*, which contradicts both (3) and (4). Perhaps, it is better to call it something like "raw fitness" to distinguish from μ.

8) In Fig. 3 it would be better, but not necessary, to replace V_{ip} with V_{ip}^t in the second line (ii).

9) There is an ambiguous usage of the word “genetic” in the manuscript. This ambiguity stems from calling x_i genes despite them not having anything to do with J_{ij} that represent the genome. Wouldn't it be better to call them metabolites, proteins, or gene products? There are also instances of a lost meaning. For example, what should one conclude from the claim on lines 242–247, especially from “the host population is both *genetically* and phenotypically *homogeneous*”? Did the authors mean that all J_{ij} are similar in the population (the term “isogenic” is used for this situation in other parts of the manuscript)? Then wouldn't it contradict the expectation of a cryptic spreading of genomes inside the neutral network of J_{ij} that tolerate mutations due to the mutational robustness? If not this, than what was meant there?

10) Another persisting ambiguity is related to the term “host type”. In some parts of the manuscript, for example on lines 166 and 388, the host type means an organism in a population. In other parts of the article the host type means H_i, that is a class of values of first three x_i.

11) I understand that $k = l_{\text{out}}$. k is used from line 90 to line 97 and nowhere else. Everywhere else l_{out} is used in place of it, including the end of the very same paragraph. k should be consistently replaced by l_{out}.

References

[1] Kaneko, Kunihiko. ”Evolution of robustness and plasticity under environmental fluctuation: Formulation in terms of phenotypic variances.” Journal of statistical physics 148.4 (2012): 687-705.
