# Supplementary material for: Evolution of phenotypic fluctuation under host-parasite interactions
Source: PLoS Comput Biol. 2021 Nov 9;17(11):e1008694. doi: 10.1371/journal.pcbi.1008694 (PMC8604345; doi:10.1371/journal.pcbi.1008694)
Supplement: Attachment — Submitted filename: Response to reviewers.docx [file pcbi.1008694.s003.docx]

**Response to reviewer 1**

We would like to thank the reviewer for the positive and insightful comments on the manuscript. We have provided our responses to the issues raised in the review (printed in italics).

***The authors study a model of host-parasite coevolution, in which hosts***

***can escape parasite attack by changing the expression pattern of a set***

***of "target genes", which however, may have fitness costs in terms of***

***reduced growth rate. Gene expression patterns depend on an underlying***

***gene-regulatory network, which is subject to mutation and selection,***

***and additionally is influenced by developmental noise (random***

***fluctuations in the network). The authors investigate under which***

***conditions gene expression becomes more or less robust to both genetic***

***changes and noise. If noise is inherently strong, its effects can be***

***enhanced by selection from parasites, leading to "noise-induced***

***fluctuations", which reduce prey growth rate but also reduce parasite***

***attack.***

***While the model and the underlying questions are interesting, I cannot***

***recommend acceptance of the manuscript in its present form, and the***

***required revisions would essentially lead to a new paper. I detail my***

***criticisms (and other comments) below.***

We thank the reviewer for pointing out our misleading descriptions and insufficient explanations.

***1) The writing is not of sufficient quality. For some parts of the***

***paper, I make rather detailed suggestions below, but I did not have***

***the time or energy to do it for the whole manuscript.***

***2) Many details of the model description are unclear, and the***

***notation is in parts not sufficiently precise (see detailed comments***

***below). In particular***

***- It is not clear how the simulations are conducted. Hosts appear to***

***be modeled as individuals (with cell divisions and a maximal***

***population size $N$), whereas predators are just modeled by a system***

***of differential equations. How are these two aspects combined?***

Thank you for these comments.

The evolution of hosts is modelled by the individual level with a fixed population size akin to the Moran process. We need an individual-based model for hosts because the phenotype is determined as a gene expression dynamics; even for isogenic hosts, the phenotypes can vary, which is an important issue to be addressed. In contrast, the degree of attack of each parasite is uniquely determined by its genotype 000,001,…,111, and therefore, only the population of each type matters. Thus, we consider only the population dynamics of each parasite type, which are given by the population growth caused by the attack to the hosts, and the mutation to the neighboring types represented by diffusion. Following your remark, we added the description of the model in the first paragraph on page 5.

***Also, how is the noise-term integrated into the differential equation for***

***the gene-regulatory network?***

Thank you for this question. We used the standard Langevin equation for the gene expression dynamics, i.e., we introduced the noise term as the last term of Eq. (1). We explicitly stated this in the first paragraph on page 4.

***What are the relevant timescales (e.g., how long is one generation)? Does the host network typically reach a***

***steady state? If yes, how fast?***

Thank you for your comment. Based on your remark, we added the description of typical timescales explicitly (one generation time of the host and the time to reach the steady state) as the first paragraph in page 5. Within this time scale, the host gene expression reached a steady state. We added a remark on this point in the first paragraph on page 5.

***Initial conditions are poorly specified. Apparently, the initial***

***gene-regulatory network is random with a fixed proportion of***

***non-zero interactions, but this is not likely to be realistic.***

Although we started from a random network, within dozens of generations, the GRN evolved to have a high-fitness expression pattern. Indeed, the four phases (regimes) are characterized by the steady state of the host–parasite system (e.g., variances and fitness). The state and quantitative measures are reached independently of the choice of initial networks. We stated this point in the first paragraph on page 9. Further, in Fig. 6, we showed that the final variance (Vip and Vg) does not depend on the initial choice.

***Also,  in many Figures, simulations for different parameter combinations***

***start at different points, but no explanation is given. It is then***

***very hard to interpret the results. For example, what does it mean***

***if V_g decreases from a initial condition which is impossible to***

***understand?***

The GRNs that initially went extinct because of parasite interactions are excluded from the sample. Therefore, the value of each variance in the initial condition is different in terms of the parameters. After sufficient generations, the variances reach stationary values. According to your comment, we added the description of initial conditions in the first paragraph on page 7.

***Another important point for clarification is the exact definition of***

***the variance component $V_g$. It seems to be a measure of***

***evolvability (rather than standard genetic variance),***

***that is, the variance that can be created by one-step***

***mutations. Making this completely clear is absolutely crucial.***

Following your remark, we removed the misleading description in the Method section on page 17 and added the correct explanation in the revised manuscript. We stated explicitly how we measured Vg in this revised explanation, which corresponds to evolvability, as you have correctly noted.

***In addition, the authors should probably add a simple measure of***

***genetic variation, that is, which part of the observable phenotypic***

***differences in the population at a given time are due to genetic***

***differences?***

Indeed, we computed Vip and Vg distinctively. The former is the phenotypic variance caused by noise for identical networks (isogenic individuals) and Vg is attributed to the genetic variation. The total variance is given by the sum of Vip and Vg, and this is explained in page 6.

***Ideally, the simulation code should be made available on, e.g.,***

***Dryad or Github.***

We will upload the simulation code on Github.

***The biological terminology is imprecise. In particular, I do not***

***like the use of the term phenotypic plasticity for noise-induced***

***phenotypic variation. For me, plasticity implies reaction to an***

***environmental cue (the reaction-norm concept). Random variation***

***without cues is better called bet-hedging (as mentioned in the***

***Discussion).***

We removed the misleading terminology “phenotypic plasticity” and replaced it by the term noise-induced phenotypic variation. However, phenotypic plasticity and phenotypic fluctuation caused by noise are correlated as demonstrated by several studies （Ref[21,23] in the manuscript）.

***Also, plasticity should not be confused with***

***evolvability (or variability), and evolvability and robustness are not***

***opposites (a phenotype can be robust, yet evolvable). Finally, one***

***might distinguish between mutational and developmental robustness.***

We removed the confusing terms and distinguished the two robustness values. Robustness against noise and mutation are distinct and defined differently; however, after the evolution of the GRN, the robustness of a certain phenotype or fitness against noise (i.e., their insensitivity to noise) often leads to the mutational robustness of the phenotype or fitness, thereby resulting in a smaller changeability of the phenotype or fitness by mutation as demonstrated in an earlier study. Ｗｅ revised the introduction to reflect these points.

***A key result is the definition of four "phases" (which I would***

***rather call regimes) regarding the evolution of the components of***

***phenotypic variance, depending on whether the parameters c (impact***

***of parasites) and sigma (developmental noise) exceed certain***

***thresholds. This is a useful conceptualization, but the results are***

***not easily seen in the Figures. A simple diagram (see below) might be***

***useful. Now, thinking about the results, the following points come to***

***mind:***

Thank you for pointing this out. We removed the misleading terminology and the term phases and replaced them by the regimes.

***In phase I, it is interesting that V_g does not decrease a lot (l.***

***234). Does this mean that selection for mutational robustness is***

***weak?***

Yes. There is a certain threshold noise level beyond which the evolution of robustness progresses. Below this threshold, mutational robustness is weak, and we added a remark about this in the second paragraph on page 9; this point was also discussed in the earlier paper without parasites [17], and therefore, we do not discuss this point in detail.

***In phase II, there is selection for developmental robustness, which***

***apparently also induces genetic robustness. This is probably not***

***surprising, since both mutations and stochastic gene expression***

***affect the same gene-regulatory network.***

The relationship between the robustness to noise and mutation during the evolution under the noise in GRNs has already been investigated in a previous study; Vip and Vg are correlated through the evolution of GRNs.

***In phase III, apparently noise is just too weak to have a***

***significant effect, so genetic variation is the only way of escaping***

***the parasite. It would be could to detail both existing genetic***

***variation (how many genotypes are present) and mutational robustness***

***(or absence thereof), that is, what is the effect of mutations.***

Following your remark, we added the description of the existing genetic variation in the second paragraph on page 11. Since mutational robustness does not evolve in region III, mutants with different gene expression levels (fitness) continually produced from the original population appear.

***The most interesting result pertains to phase IV: Noise is important***

***and its effects seems to be amplified by selection. In addition, V_g***

***goes up, too (line 262), mirroring the result for phase II. However,***

***Fig. 5 shows that high sigma leads to decrease in V_g. Does this***

***mean that there is selection for genetic robustness but***

***strongly stochastic development, or is there just less selection to***

***increase sensitivity to mutation? Again, without reasonable initial***

***conditions, this is hard to tell.***

The evolution of mutational robustness and developmental robustness occurs even if the interaction with the parasite is sufficiently strong, as long as the noise level is above the threshold. For example, the variance of the output genes in Fig. 6 decreased with evolution. The hosts deal with parasites by using phenotypic variance by noise Vip, while maintaining the robustness to the mutation to decrease Vg. This behavior is always observed after evolution, and it is independent of initial conditions. Following your remark, we added some explanation on this point on page 9. Further, the evolved state after sufficient generations does not depend on initial conditions. As an example, we plotted the results from two distinct initial conditions in revised Fig.6 (i) vs (ii).

***This is probably difficult, but ultimately, V_g and V_ip (and their***

***evolvability) are properties of the gene-regulatory network, which***

***however, is treated as a black box. Can one say anything about the***

***properties of the network that lead to a given result?***

We studied the proportions of typical network motifs; however, the differences between the regions were small. The motifs that activated the output genes increased, whereas those that inhibited them decreased. This network analysis suggests that robustness to noise occurs as a result of the regulation among many genes. Following your remark, we added the description of existing genetic variation in the third paragraph on page 15.

***Two other studies that have looked at the evolution of***

***plasticity in the context of predator-prey interactions:***

***- Yamamichi, M., Yoshida, T., & Sasaki, A. (2011). Comparing the***

***effects of rapid evolution and phenotypic plasticity on***

***predator-prey dynamics. The American Naturalist, 178(3), 287-304.***

***- Svanbäck, R., Pineda-Krch, M., & Doebeli, M. (2009). Fluctuating***

***population dynamics promotes the evolution of phenotypic plasticity.***

***The American Naturalist, 174(2), 176-189.***

***And some studies about genetic adaptation vs. plasticity or***

***bet-hedging:***

***- Svardal, H., Rueffler, C., & Hermisson, J. (2011). Comparing***

***environmental and genetic variance as adaptive response to***

***fluctuating selection. Evolution: International Journal of Organic***

***Evolution, 65(9), 2492-2513.***

***- Botero, C. A., Weissing, F. J., Wright, J., & Rubenstein, D. R.***

***(2015). Evolutionary tipping points in the capacity to adapt to***

***environmental change. Proceedings of the National Academy of***

***Sciences, 112(1), 184-189.***

Following your remark, we have added the above references.

***Specific comments:***

Thank you for pointing out the typographical errors and the minor grammatical mistakes. We have corrected the text following your suggestions.

***- 81: are gene expression levels between 0 and 1?***

Yes, the gene expression levels are between 0 and 1.

***- How is the white-nose term eta incorporated in a differential***

***equation. Is equation (1) supposed to be a stochastic differential equation***

***(but eta is not Brownian motion)?***

We considered the standard Langevin equation for the gene expression dynamics.

***- Do gene expression levels of an individual cell typically approach a***

***steady state (characterized by x = f + epsilon + I), despite ungoing***

***white noise? This is hinted at in the Methods section, but***

***should be stated more clearly. Also, does this mean that the***

***stochastic term mainly plays a role at the beginning of the life***

***cycle of a cell (when all x_i's have been reset to small values)?***

The division time of the host is determined by the individual growth rate that ranges between 500–1,000 s. The network of hosts reaches a steady state around 200 s. Following your remark, we added the description of this point in the second paragraph on page 5.

***Also, are gene expression levels restricted to 0..1 (as indicated in***

***line 120)? What if an input gene with $I_i = 1$ receives additional***

***input f = 1?***

We have corrected our typographical error in equation (1). Since the input acts on some input genes, I_i is inserted into the sigmoid functions f(z).

***$t$ in equation (1) is the timescale of the dynamics of the gene***

***regulatory network. What is the timescale of population dynamics?***

***(How long is one generation, on average)?***

Following your remark, we added the description of the timescales of population dynamics in the first paragraph on page 5.

***$k$ is called $l_{out}$ elsewhere.***

Following your remark, we removed $k$ and replaced it by $l_{out}$.

***Please explain the notation of the equation at the end of the***

***line, and what it means biologically (absence of correlation between***

***genes?). Do the angled brackets***

***indicate a inner product? Is $\delta_{ij}$ a Kronecker delta? What***

***is $t'$? And what is the difference between $\delta$ with two***

***indices and with an argument in parentheses?***

We explained the notation at the end of line, with its biological meaning.

Here, the noise is applied to the expression of each gene and it is not correlated across genes. Such noise is attributed to the stochastic gene expression that originates in the fluctuations in chemical reactions with a small number of molecules. The angled brackets indicate the covariance $E(\eta_{i}(t)\cdot\eta_{j}(t))$. $\delta_{ij}$ is a Kronecker delta, and $\delta(t-t’)$ is a Dirac's function E(w(t1)⋅w(t2)). Following your remark, we added the description of this point in the first paragraph on page 4.

***N has not been defined at this point.***

Following your remark, we added the description of the total number of host populations in the first paragraph on page 4.

***I don't think it is correct to say the $\mu_0$ is maximal***

***fitness, since the sum does not seem to be restricted to values***

***between 0 and 1.***

Following your remark, we removed the misleading terminology. We removed the term maximal fitness and replaced it with basal fitness.

***The $i_j$'s are allelic values, not parasite genotypes. A***

***parasite genotype is a sequence $i_1 ... i_{l_p}$. Also, the end of***

***the sentence $such that $\{0, 1\}^{l_p}$ seems to be missing***

***something.***

Following your remark, we deleted the sentences with $\{0, 1\}^{l_p}, and we explained the parasite types explicitly.

***I would speak of parasite genotypes, not species, since they***

***connected by mutation.***

Following your remark, we removed the confusing terms. Instead, we adopted the term “parasite genotypes.”

***and elsewhere: Also, I would prefer to call the term defined by***

***equation (3) and (intrinsic) growth rate, whereas fitness is defined***

***by equation (4).***

Thank you for pointing this out. We adopted the term raw fitness for $\mu$ defined by equation (3).

***Equation (4): So, $P_m$ is the density of the parasite that can***

***attack host $i$?***

Yes. $P_m$ is the population density of the parasite that matches with the host $i$. We stated this point in the third paragraph of page 4.

***Where does the $k$ come from? Is it also randomly selected?***

We randomly select a gene $k \neq i, \neq j$. Then, the path j->i is replaced by i->k to represent the mutation of a path in GRN. We added the description on this point in the first paragraph on page 5.

***It should be $l_p$ in the denominator of the second equation.***

$l_p$ is a binary string length, and therefore, there are ${Np=2}^{l_{p}}$ parasite genotypes.

***I don't think one can use "distribution" the way***

***you do it. What you probably mean is that $x_i$ has a probability***

***distribution, that is, it is a random variable. But is is enough to***

***say "...which leads to random variation in the expression of $x_i$".***

Following your remark, we corrected the text following your suggestions.

***Isn't $v_i^{(l)}$ the same as*** ***$V_{ip}^{(l)}(i)$ in line 164?***

Yes. We have replaced $v_i^{(l)}$ by $V_{ip}^{(l)}(i)$i.

***Even with the details in the Methods section, the definition of***

***$V_g$ is not clear. Apparently it is not simply the between-genotype***

***component of phenotypic variation (the variance of the $\bar***

***x_i^{(l)}$, but rather some measure of genetic variability or***

***evolvability, that is, the variance in gene expression levels***

***between genotypes created by single mutations (specific to***

***a given focal genotype). It is very important to make this***

***definition clear and unambiguous. Below (editorial comments to line***

***391) I have tried to reformulate the Methods section in accordance***

***with my interpretation of what you mean by $V_g$.***

Following your remark, we removed the misleading description in the Method part in page 17 and added a clear description.

***that is, you are average across both genotypes $l$ and genes***

***$1$ to $l_{out}$?***

Yes. We averaged the variance across the output genes.

***Table 1: $Is $I_j = -5$ a typo?***

Thank you for pointing out our typographical error. We corrected the text following your suggestion.

***Table 1: $T_p$ is not mentioned anywhere in the manuscript. Is this***

***a type for $D_p$?***

Thank you for pointing out our typographical errors. We have corrected the text following your suggestions. $D_p$ denotes the mutation rate of the parasite. We added the correct explanation of $T_p$.

***What is sigma in Fig. 1?***

Sigma is zero. Following your remark, we added the description on this point in Fig. 1.

***It seems that all the frequent phenotypes have at most one***

***target gene switched off. This makes sense since changing the***

***expression of a single gene is enough to escape any particular***

***parasite. Is this pattern more general?***

Yes. The expression of a single gene is sufficient to escape any particular parasite. This is a general trend in the parameter areas we studied.

***This argument for c_t = 1 works because $P_m$ is less than or***

***equal to $1$***

Yes. $P_m$ is 1 if it most closely matches with the host.

***Fig. 2: Since the variances are averages over time, can you add***

***error bars? Same for Fig. 5.***

Following your remark, we added error bars in Figs. 2 and 5.

***- Fig. 3: Why do the variances vary between parameter combinations***

***even at t = 0? What were the initial conditions? Without an aswer to***

***these questions, the results are hard to interpret.***

GRNs that initially went extinct as a result of parasite interactions are excluded from the sample. Therefore, the phenotypic variance at the initial conditions depends on the parameters $c$. Following your remark, we added the description of the initial conditions in the first paragraph on page 7.

***Before defining the four phases/regimes, the critical value***

***$\sigma_c$ should be introduced (maybe by immediately referring to***

***Fig. 4+5; see my suggestion in editorial comments below).***

***I suppose this value is purely heuristic, or is***

***there some analytical justification? I also have to say that I find***

***it difficult to see the four phases when looking at Fig. 4 (see my***

***next point below), and that in Fig. 5, sigma_c might also be placed***

***at 0.02 (or is it the beginning of the decrease in V_g you are***

***focusing on?).***

Following your remark, we corrected the text of the critical value $\sigma_c$ in the first paragraph on page 9. This value was determined heuristically at the point where Vg^t and Vip^t (target genes) are comparable. We replaced the value of $sigma_c $ with 0.015.

***- I think it would be helpful to add a simple plot showing the***

***parameter ranges pertaining to the four phases: Just a coordinate***

***system sigma vs. c with a horizontal line and c_t, a vertical line***

***sigma_c and roman numerals I, II, III, IV in the respective areas.***

We have revised Fig.4, according to your suggestions.

***But is Fig. 1 for sigma < sigma_c?***

Yes. In Fig.1, we used sigma = 0.0(< sigma_c), as stated in the caption.

***Do you mean c < c_t?***

Thank you for pointing out our typographical error. We corrected the text following your suggestion.

***So, V_g remains high, but phenotypic diversification is not***

***based on genetic diversification? This is somewhat confusing, even***

***if V_ip > V_g. What seems more significant is that, according to***

***Fig. 5, V_g seems to be repressed when sigma is high, even if***

***phenotypic diversity is favored.***

Genotypic diversification occurs even in region Ⅳ. However, the degree of diversification is lower than that in region Ⅲ, as shown in Fig. 5.

***Somewhere (probably earlier than here), you should remind the***

***reader that initial values of V_g are high because initial networks***

***are random.***

Following your remark, we have added the description of this point in the first paragraph on page 9.

***Fig. 4: How exactly are the tics at the axes related to the colored***

***squares. For example, is the bottom-left square for c = sigma = 0?***

The tics at the axes corresponded perfectly with the colored squares. The bottom-left square is the condition where c = 0 and sigma = 0.001. Following your remark, we added the description of the tics in Fig.4.

***In Fig. 5, V_g = V_ip for sigma = 0.02 and c = 3, yet in Fig. 6,***

***which is also for sigma = 0.02, when switching c from 0 to 3, not***

***much happens for the output genes, whereas for target genes, the***

***system evolves further away from the diagonal (as V_g increases***

***also).Can you add a plot for target genes only to Fig. 5?***

Following your remark, we added the value of Vip and Vg for target only. For target genes, Vip exceeds Vg at around sigma = 0.015. The system evolves further away from the diagonal to increase the fluctuations.

We also replaced Fig.6 to include the result of evolution after the switch from c = 0 to 3.

***In addition, to complement Fig. 4 (whose color code is compact but***

***not easy to read if one wants to see the details), I think it would***

***be could to add a Figure to the supplementary material which shows***

***Figures as in Fig. 5 (for both target and output genes) for***

***different values of c.***

Following your remark, we added a new figure in the supplementary material.

***Also, when looking at Fig. 4 in the c direction, it seems that most***

***of the increase in V_p (see Fig. 2) is due to increase in V_ip, not***

***V_g.***

As you have correctly pointed out, the increase in Vp is mostly because of V_ip ($\sigma>\sigma_c$). However, as shown in Fig. 5, for a small sigma, Vg is dominant over Vip. In regionⅠ, robustness evolution to mutation does not occur, and therefore, Vg is large even when c = 0; however, Vg is evolved to increase from the initial value (see Fig.3 c = 2, sigma = 0.01 case).

***What were the initial conditions for Fig. 7? Why are there***

***different initial conditions between c = 0 and c = 3 and between***

***target and output genes?***

GRNs that initially went extinct because of parasite interactions are excluded from the sample. Therefore, the initial conditions for c = 0 and c = 3 are different, and both V_g and V_ip tend to be larger at c = 3. Following your remark, we added the description of this point in the third paragraph on page 12.

***I am not quite sure what to make of this paragraph. What I***

***can see in Fig. 7 is that, for c = 0, V_ip and V_g decrease in both***

***target and output genes (indeed, there should be no difference***

***between the two sets), even though V_g shows an initial increase***

***(which is not explained, and might not be terribly important). In***

***contrast, for c = 3, V_g for target genes increases rapidly,***

***followed by a small decrease in V_ip. For non-target output genes,***

***V_ip decreases notably, and V_g decreases slightly, but less so than***

***for c = 0. Interpretation is complicated by the fact that the***

***different initial conditions are not explained. Also, I'm not sure***

***about the notion of correlated evolution, and how to see when the***

***correlation is stronger or weaker. As long as evolution of both***

***variances is more or less directional, the trajectory will always***

***resemble a line (as opposed to a cloud). It is interesting thought***

***that evolution of expression variation in target and non-target***

***output genes is at least partially decoupled, indicating a high***

***flexibility of the genetic networks.***

Following your remark, we changed the layout of our manuscript.

First, we discuss the results of starting with initial random networks in Fig. 6(i). Here, Vip and Vg increase rapidly, while Vg<Vip is maintained. For the non-target output genes, both variances evolve to decrease, and as you have correctly pointed out, the output genes and target genes are partially decoupled. We added the description of this point in the first paragraph on page 13.

Next, we investigated whether a similar evolution with increased fluctuations could occur in response to the introduction of parasites, even after phenotypic and genotypic robustness have evolved in Fig. 6 (ii). Fig. 6(i) and (ii) shows that variances in the gene expression of GRNs finally evolved into the same value, while maintaining the phenotypic fluctuations. We added the description of this point at the second paragraph in page 13. Both variances are decreased in a correlation, which has been investigated in previous studies [17,24]; however, the fact that they are correlated in the direction of the increase is not an obvious phenomenon. It was found for the first time as a result of the evolutionary simulation in this study.

***Figure 8 and accompanying text: It does not become clear here (but***

***only in line 354) that the fitness costs are higher for noise than***

***for genetic variation (probably because noisy system tend to switch***

***of more output genes than necesssary to escape parasites). This***

***should be made clear.***

Because other output genes tend to be turned off as a result of the fluctuation of the target genes, there is a trade-off between Vip and raw fitness. With the phenotypic variation caused by genetic variation, their raw fitness is not decreased considerably. In regime Ⅲ, the expression of target and non-target output genes are almost decoupled, and therefore, the variation in the target-gene expression does not turn off the other output genes.

Following your remark, we added the description of this point in the fourth paragraph on page 13.

***Fig. S5: How is gamma the timescale of parasite dynamics? It figures***

***in equation 1 and, in my view, defines the timescale at the which***

***the host gene expression is determined by the GRN.***

Gamma corresponds to $T_p$, which is the generation time of the parasite.

Following your remark, we removed the misleading description Gamma in Fig.S5 and added the parasite generation time $T_p$.

***Editorial comments:***

***------------------***

***- Abstract: What do you mean by "Through the evolution of a given***

***environment"? Maybe simply: In a constant environment, ...***

***- 13: as discussed?***

***- 30: Even after evolution in a constant environment,***

***- 33: potential***

***- 33: This leads to the following questions:***

***- 34: Why are phenotypic fluctuations not reduced further***

***- 38: In natural populations***

***- 79: of the genotype-phenotype map***

***- 81: each other's expression***

***- eq. (1): use larger parenthesis in equations where appropriate;***

***e.g., in Latex, this would be $\left( ... \right)$.***

***- 94: the path density, that is, the fraction of non-zero values of***

***$J_{ij}$.***

***- 113: $N_p$ parasite genotypes***

***- 128: If total cell number exceeds an upper limit N, ...***

***- 155: The model includes a noise component that allows gene***

***expression levels x_i to vary even among individuals sharing the***

***same gene regulatory network.***

***- 158: over the entire host population***

***- 159: In addition***

***- 159: of the gene expression level $x_i^{(l)}$ within each host type***

***$l$.***

***- 160: Because of the noise term $\sigma \eta_i$ in equation (1),***

***- 161: this variance is positive***

***- 162: This sentence might be better placed after the one in line 158.***

***- 165: The variance of gene expression expression levels in an***

***isogenic [I would probably say monomorphic] population***

***- 167: due to genetic differences***

***- 179: the host population is dominated by gene regulatory networks...***

***- 181: This sentence is redundant***

***- 189: less preferred/less fit***

***- 190: parasites induce negative frequency-dependent selection***

***- 191: parasite genotype distribution***

***- 196: with polymorphic genotypes (i.e., networks $J_{ij}$)***

***- 198: This sentence can be omitted***

***- 199: The Figure shows a marked increase in phenotypic variance at a***

***threshold interaction strength $c = c_t \approx 1$. [The following***

***sentence can be omitted.]***

***- 202: within the population***

***- 223ff: Maybe rewrite along the following lines: "Fig. 3 shows***

***evolutionary time courses for the variance components V_ip and V_g***

***(for both output and target genes) for various combinations of c and***

***sigma. More systematic results regarding steady-state values are***

***shown in Fig. 4 and 5. In addition to the critical interaction***

***strength c_t, we can see (e.g., from Fig. 4 and 5) a critical noise***

***level $sigma_c \approx 0.01$ (or $0.02$?), above which V_ip***

***increases markedly. Based on these two thresholds, the results can***

***roughly be classified into four phases/regimes: ...***

***- 229: I would replace "phase" by "regime" throughout***

***- 230: When the noise is below a threshold of $\sigma_c = 0.01$ and***

***the interaction is weak ($c < c_t = 1$), $V_{ip}$ is naturally low,***

***whereas $V_g$ is maintained at a high level (Fig. 4), indicating***

***that mutational robustness does not evolve. Host phenotypes (i.e.,***

***gene expression) fluctuate around the fittest type, as shown in Fig***

***1( i ), where most of the output genes are expressed, whereas the***

***variances are sustained at moderate values owing to genetic***

***variation in network structure.***

***- 234: not particularly robust against mutations***

***- 242: I would remove the sentence "As V_g is small"***

***- 244: to both noise and genetic change***

***- 251: V_g remains at high levels***

***- 256: I would remove the sentence "For each host phenotype..."***

***- 260: As exemplified by the case...***

***- 389: among those***

***- 390: due to genetic variation***

***- 391ff: Here is an attempt to clarify this part: $V_g^{(l)}(i)$ is***

***defined as the phenotypic variance created by mutation in genotype***

***$l$ [my interpretation, but else, how would it depend on $l$?]. We***

***first computed $\bar x_i^{(l)}$, i.e., the average of $x_i$ over***

***noise, for each genotype $l$. Then, we computed the variance of***

***$\bar x_i^{(l)}$ in a heterogenic [polymorphic] population created***

***by adding random one-step mutations to the interaction matrix***

***$J_{ij}^{(l)}$ of this genotype. More precisely, in the simulation,***

***there are up to $N$ individuals with different genotypes***

***$J_{ij}^{(l)}$ , with each potentially having a different gene***

***expression pattern. To calculate the $V_{ip}^{(l)}(i)$ and***

***$V_g^{(l)}(i)$, $L$ individuals (genotypes?) are selected at random***

***from the population. For each genotype individual (with genotype***

***$l$), we created $N_c$ clones. Then, we computed the phenotypic***

***variance (i.e., variance in gene expression) for each gene over each***

***clonal population at $t = 1000$, after gene expression reaches a***

***steady state. This gives rise to values $V_{ip}^{(l)}(i)$, that is,***

***the noise-induced variance of gene $i$ for genotypes $l$.***

***Subsequently, we created genetic variation in $J_{ij}^{(l)}$ by***

***adding a single random mutation to the network of each of the $N_c$***

***clones of genotype $l$, enabling $V_g(i)^{(l)}$ to be estimated as***

***the variance of the $\bar x_i^$ for each clone. We used $L = 30$ and***

***$N_c = 50$ for all simulations.***

***- 205: This critical interaction strength $c_t \approx 1$ follows from***

***the fitness function in equation (4). Host growth rate is maximized***

***when the expression level of all the output genes is close to $x_j =***

***1$. Switching off one of the target genes reduces the maximal growth***

***rate by one unit. This fitness loss is compensated by the reduced***

***risk of parasitism if $c P_m > 1$. And since, $P_m \leq 1$ (with the***

***density of the most frequent parasite often close to $1$), the net***

***fitness gain is positive if $c \gtrsim 1$. Indeed, Fig 2 shows that,***

***for $c > c_t$, the variance increases substantially. When the***

***interaction is strong (e.g., $c = 4$), the number of host phenotypes***

***approaches the maximum number, $2^{l_p}$ (= 8), corresponding to all***

***possible parasite species (see Fig 1(iii)).***

***- 215: new paragraph; omit the first sentence; then "In our model,***

***adaptive phenotypic diversity can have two different origins..."***

***- Fig. 5, caption: sigma_c, not sigma_th; also, why are you mixing***

***$\approx$ and $\sim$ (double and single "wave")***

***- 272: indicating that there is little robustness to mutation, and***

***diversity of target gene expression levels is based primarily on***

***genetic variation. In contrast, when the noise level exceeds***

***sigma_c, V_ip > V_G and diversification of gene expression patterns***

***is achieved mostly through phenotypic plasticity. [Remove the***

***following sentence.]***

***- 280: In the absence of parasites, the variances V_ip and V_g***

***decrease as the host phenotype adapts to the environment, indicating***

***evolution of mutational and developmental robustness (phase II).***

***Next, we investigated whether, once such robustness as evolved,***

***evolution of plasticity is still possible in response to the***

***introduction of parasites.***

***- 284: had decreased from their high initial values***

***- 287: before increasing again***

***- 288: show a notable increase within 100 generations. This increase***

***is most notable in target genes, but can also be detected for the***

***other output genes. Thus, notwithstanding prior evolution of***

***robustness, plasticity can still evolve in response to parasite***

***infection.***

***- 293: Correlation between V_g and V_ip?***

***- 294: Why compare to a previous study? Figure 7 shows evolution in***

***the absence of parasite interactions in the present study.***

***- 299: Closer than what?***

***- Fig. 7: Axes labels are missing; end of caption should probably red***

***(circles in (a)).***

Thank you for pointing out these typographical errors. We have corrected all of them.

We believe that the present revision throughout the manuscript will mee all of your requests. We would be grateful if you consider the publication of the present version.

**Response to reviewer ２**

We thank you for your positive and insightful comments on the manuscript. We have provided our response to the issues raised in the review below (printed in italics).

***The Fitness and reproduction part describes mutations during the reproduction (line 132), but it is not written how often they were added to Jij . There is no clear parameter that controls the mutation rate in Table 1. Instead, there is a parameter Tp named “evolutionary speed” with no explanation of what it is. Perhaps it is somehow related to the mutation rate but this stays obscure. The same problem 2 exists for the parameter Dp, the mutation rate of the parasites. It is neither listed in Table 1 nor its value is revealed anywhere in the text.***

Following your remark, we removed the misleading description in the Model part and added the correct explanation. We stated the parameters $T_{p}$ and $D_{p}$ in Table1.

***It is not clear how exactly to compute V (l) ip . First, the term “host-type” (line 388) should not be used for the l-th organism in the population, as it brings confusion (see below in the minor comments). Second, when V (l) ip is computed is it that only (1) is numerically solved for the same Jij but different initial x and noise realisations, or is it that (1) is solved along with the simulation of the population growth by (2), but with suppressed mutations and no parasite attack, or something else? It is not clear from the description. The two situations differ in the presence or absence of the random re-initialisation of the dynamics at a random initial datum in (1) after each division event in (2), which can lead to a situation when at large time the population has a substantial fraction of unsettled in terms of their internal metabolic state individuals. Likewise it is not clear how to compute V (l) g : with respect to only metabolic simulation (1) or the full population simulation (1)–(2).***

Following your remark, we removed the misleading description in the Method part and added the correct explanation. We stated explicitly how we measured $V_{ip}$ and $V_{g}$ in page 17. For both variances $V_{ip}^{\left( l \right)}$ and $V_{g}^{\left( l \right)}$, we took $J_{ij}$ along the simulation. Then, equation (1) is numerically solved for the same $J_{ij}$ without the parasite dynamics under noise. By repeating the simulations over 50 samples for different random numbers for the noise, we obtain the distribution of $x_{i}$ and the average $\bar{x_{i}}.$, and its variance. This variance gives $V_{ip}^{\left( l \right)}$. Then, by substituting the mutation to $J_{ij}$, we computed the variance of $\bar{x_{i}}$, which leads to $V_{g}^{(l)}$.

***There is a contradiction in the value of the external input Ij . On line 103 it is indicated that Ij = 1. In Table 1 it is indicated that Ij = −5. Obviously, from these two only 1 can represent the real value. What was used in the actual numerical experiment?***

Following your remark, we removed the typographical error in Eq. (1) and added the correct explanation. $I_{j}$ denotes the external input for “input genes,” and $I_{j}=1$ is correct.

***How are the parasites Pi initialised?***

Following your remark, we added the description of parasite dynamics explicitly at the first paragraph in page 6. Initially $P_{i}$ is equal over all types with $\sum P_{i}$. However, the results are independent of the initial parasite distribution.

***(1) There must be a typo in the indices of J-s at line 135 describing the mutation process that conserves α. Right now the description is nonsensical.***

Thank you for pointing out our typographical error. We have corrected it following your suggestion.

***(2) Line 92. θi is not simply randomly selected but from a uniform distribution, isn’t it? The same goes for xi during the re-initialisation after a division.***

The value $\theta_{i}$ is distributed randomly between fixed limits of 0.05 and 0.3. $\theta_{i}$ is randomly selected from a uniform distribution. We stated these in the revised text.

***(3) Line 93. Please, elaborate why*** $\sqrt{\boldsymbol{\alpha M}}$ ***is the correct scaling factor. It is not obvious for me.***

Because the connection is sparse, many $J_{ij}=0$. The average number of inputs for the gene is $\alpha M$. We normalized by the square root of the size number, and therefore, the variance of $J_{ij}$ is independent of $\alpha M$ when $J_{ij}$ is randomly chosen. This square root scaling is commonly adopted in spin glasses and gene regulation networks.

**(4) In my taste exp, linp, lout better look as exp, linp, lout. Of course, this is a matter of taste, not a requirement.**

We revised the text following your suggestion.

***(5) Line 109. What does “ds” mean? Must be a typo.***

Thank you for pointing out our typographical error. We corrected it following your suggestion.

***(6) Line 143 and line 159. Almost the same notation (vi and υi) is used for two different entities. Furthermore, v (l) i introduced on line 159 (the variance of the gene expression of x (l) i ) is never used in the text after that place. Was it a typo?***

Thank you for pointing out our typographical error. We replaced $v_{i}^{(l)}$ by $V_{ip}^{(l)}(i)$.

***(7) Fig. 8. On this figure and in its caption the quantity*** $\sum_{\boldsymbol{i}}^{\boldsymbol{l}_{\boldsymbol{out}}} \boldsymbol{x}_{\boldsymbol{i}}$ ***is called fitness, which contradicts both (3) and (4). Perhaps, it is better to call it something like “raw fitness” to distinguish from µ.***

Thank you for pointing this out. We adopted the raw fitness following your suggestion.

***(8) In Fig. 3 it would be better, but not necessary, to replace Vip with V t ip in the second line (ii).***

Following your remark, we replaced $V_{ip}$ with $V_{ip}^{t}$ in Fig.3.

***(9) There is an ambiguous usage of the word “genetic” in the manuscript. This ambiguity stems from calling xi genes despite them not having anything to do with Jij that represent the genome. Wouldn’t it be better to call them metabolites, proteins, or gene products? There are also instances of a lost meaning. For example, what should one conclude from the claim on lines 242–247, especially from “the host population is both genetically and phenotypically homogeneous”? Did the authors mean that all Jij are similar in the population (the term “isogenic” is used for this situation in other parts of the manuscript)? Then wouldn’t it contradict the expectation of a cryptic spreading of genomes inside the neutral network of Jij that tolerate mutations due to the mutational robustness? If not this, than what was meant there?***

$x_{i}$ is the protein expression of level from each gene $i$. As remarked in the beginning, we keep the term gene expression level $x_{i}$ because it is often adopted, especially in the study of gene expression dynamics by GRN.

Here, we are interested in the evolution of GRN, which is determined genetically. We do not discuss the acquirement of new protein by evolution. Instead, we focus on the genetic change that causes mutual regulation, which changes the expression level. Hence, we focus on the evolution of $J_{ij}$. Indeed, the fast evolution of GRN has been noted and the evolution of GRN has been extensively investigated [21].

***(10) Another persisting ambiguity is related to the term “host type”. In some parts of the manuscript, for example on lines 166 and 388, the host type means an organism in a population. In other parts of the article the host type means Hi , that is a class of values of first three xi .***

We removed the misleading terminology. We removed the term “host type” on lines 166 and 388, and instead, we adopted the host genotype.

Thank you again for all of your constructive comments. We believe that the present revision throughout the manuscript will mee all of your requests. We would be grateful if you consider the publication of the present version.
