# Supplementary material for: Evolution of phenotypic fluctuation under host-parasite interactions
Source: PLoS Comput Biol. 2021 Nov 9;17(11):e1008694. doi: 10.1371/journal.pcbi.1008694 (PMC8604345; doi:10.1371/journal.pcbi.1008694)
Supplement: Attachment — Submitted filename: Response to reviewers.docx [file pcbi.1008694.s004.docx]

We thank the reviewer for the time and effort put in towards the review of this manuscript. We have incorporated several changes based on the comments of the reviewers. The detailed responses to the reviewer comments are given below.

***Reviewer #: General comments:
-----------------***

***I am still not too happy with the revised version, and I think another
major revision is necessary. My main points of critique are the
following:
1) The writing still needs lots of improvement. It might be good if
the authors could have the manuscript checked by a native English
speaker. I make some suggestions below, but they are far from
exhaustive.
2) I think I understand the model assumptions much better now, but
some points need to be made clearer for the reader. I also think that
there is a problem with the measure V_ip, as part of this variance
does not affect fitness (see below). I therefore suggest to look at a
version of V_ip that only looks at on/off switches of genes, not
small variation in expression level.
3) I am still having a hard time understanding some of the results.
While this is in part due to the "black-box nature" of the GNRs, I
think the manuscript might be improved by (i) adding a measure of V_ip
that focuses on on/off switches (see above) and (ii) adding
information about the relation between V_ip and V_g in random
networks.
I detail these comments in the following section.***

We understand the concern raised by the reviewer. We apologize for the issues in the previous manuscript. The manuscript has been checked by an English editing service. Further we have added some explanation and figures,

following your suggestion so that the results presented here

can be understandable. Given below are point-by-point replies to

each of your comments.

***Major comments:
--------------***

***- For a single gene, if the function $f$ is assumed to be constant,
the Langevin equation (1) describes an Ornstein-Uhlenbeck process,
in which gene expression levels x_i fluctuate around a mean of f +
epsilon. More precisely, the stationary distribution is Gaussian
with mean f + epsilon \approx f and variance sigma^2/(2 gamma); this
should be mentioned in the manuscript; for the largest noise level
sigma = 0.05 (and gamma = 0.1), the variance of the OU process equals
0.0125, and the corresponding standard deviation is 0.112, meaning
that, for f close to either 0 or 1, genes can still be essentially
"switched on and off", with expression levels in the vicinity of 1
or 0 (but note that in the latter case, the actual x values can
become negative, unless this is somehow excluded in the simulations;
please clarify). Importantly, the variation around these values is
insignificant for fitness (the interaction with parasites depends
only on "rounded" or discretized values, and growth rate mu depends
on the mean expression level of output genes), even though it is
essential for the dynamics of the gene-regulatory network (i.e.,
for triggering noise-induced variation).***

**Response:** We have excluded negative values of x in our simulations. In the case where it is negative, we set the expression of x to zero. As you pointed out, there is no significant effect of noise on the fitness for parasite infection at the fixed point of x = 0 or 1. Nevertheless, the noise is important for the dynamics, and it is essential because phenotypic fluctuations are amplified in the GRN, and because noise-induced switches occur. We have added a remark on this point at the Models and on the third paragraph at page 13.

***- I think it is useful to introduce the terms mutational robustness
(which prevents changes in gene-expression patterns due to mutations
in the network structure) and developmental robustness
(which prevents on/off switches in the face of the white-noise
fluctuations in gene expression level).***

**Response:** Thank you for your comment. Based on your remark, we introduced the terms mutational robustness and developmental robustness at page 6, and used them throughout the manuscript.

***- The variance sigma^2/(2 gamma) of the Ornstein-Uhlenbeck
process (i.e., the variance of small-scale fluctuations in
expression levels) directly contributes to the measure V_ip (which
should be the sum of this variance and the variance due to on/off
switches). This blurs the interpretation of some results, and in
particular, makes it hard to see evidence for evolution of
developmental robustness. For example, in Fig. 4A, V_ip increases
with increasing sigma, but we cannot say how much of this effect is
due to the direct effect of expression-level fluctuations (increase
in sigma^2/(2 gamma)), and how much due to an increase in
noise-induced on/off switches. Similarly, in Fig. 5, the blue lines
(V_ip) beyond sigma = 0.02 appear to be roughly parallel to
sigma^2/(2gamma), which would suggest that noise-induced variation is
triggered around $sigma = sigma_c$, but that nothing much happens
beyond this value in terms of genes being switched on or off.***
***- Since for the interaction with the parasite only the on/off switches
matter, I suggest to look at a measure V_ip' that reflects only this
variation. Such a measure might be V_ip - sigma/(2gamma), the
variance of x_i after rounding to 0 or 1, or the probability
that a given gene is switched on (note that when x_i values are
discretized to 0 and 1, the resulting variance is the variance of
Bernoulli distribution, which is p(1-p), where p = P(x = 1)). If
this measure goes down (relative to the value in an unevolved
network), it is evidence for evolution of developmental robustness.***

**Response:** In order to evaluate how much the noise-induced variance is due to the switching in expression, we used the function $z=tanh\beta(x-0.5)$ with $\beta=100$ as a variable representing the on/off of the gene expression and define V_ip', as the variance of $z$. Based on your remark, we added a new figure and discussion about noise induced switch to the manuscript. The results are shown in Fig. ６ and S9. At noise strengths above sigma_c=0.02, the value of V_ip' for the target gene increases from its initial level through the evolution and maintains a high value. On the other hand, the variance of the non-target output gene decreases with the noise strength, and the developmental robustness evolution occurs. Hence the main conclusion is not changed. We have added the explanation of the result (page 14).

- ***I repeat that V_g is not standing genetic variation (this would be the
variance of $\bar x$), and in contrast to what the authors claim in
the reply letter, V_g + V_ip does not equal V_p. One way to
see this is that V_ip is expressed within a generation, but V_g only
between generations. So be careful to not call V_g the variance "due
to genetic differentiation". A population might be genetically
homogeneous and still have positive V_g.***

**Response:** As you pointed out, V_g measures the effect of genetic variation on phenotype by creating a heterogeneous population from a randomly selected network J_ij. Hence, even a clonal population can have a positive one as a result of mutation. Still, we used the same mutation rate in the evolution simulation, so that the population in each simulation is not genetically homogeneous. The term “differentiation” could be misinterpreted as you rightly pointed out, and thus, we have replaced it by variance due to genetic mutation.

***- I am still struggling to understand the initial variance values in
Fig. 3 and 6 (e.g., why is V_g so low initially in Fig. 3[i]?). You
write that genotypes that die out due to parasitism are excluded
from the sample, but do these extinctions happen immediately at t =
0?***

**Response:** We understand the concern raised by the reviewer. We have excluded the extinction samples during the first round of simulation. The presented results are after the parasite infection at the time t=0. To make this clear, we stated that the plot is obtained from the first generation rather than 0^th^ generation. Accordingly, the samples with small V_ip are excluded. As for V_g, we had previously applied additional correction to compensate this removal of low V_ip samples. We found that this additional correction made the initial Vg values in Fig. 3 and 6 in the previous manuscript artificially too low. (for the case with strong noise). Hence, we stopped this artificial correction and revised the figures. Now the initial Vg values plotted in revised Fig. 3 are of the same level, regardless of the noise strength. After evolution, this modification of initial V_g does not have a significant impact, and thus, the final result and our claim do not change.

***- In addition, you write that in a previous paper you have shown that
V_ip and V_g are correlated (I suppose this means positively
correlated?). For readers of the present paper, I think it would be
useful to repeat these results, that is, to show the relation of
V_ip (or V_ip') and V_g in random networks for various values of
sigma.***

**Response:** Thank you for your comment. Based on your remark, we added the result of the positive relation of V_ip and Vg for c=0 in Fig. S8 on supporting information and added a remark on this point on the third paragraph at page 11.

***- With the additional information mentioned above, I hope to better
understand some of the results. Currently, I still have the
following questions:
- Fig. 1(i): I don't understand why for c = sigma = 0, there are such
large fluctuations in host phenotype frequencies, with the optimal
phenotype sporadically dropping to about 50% of the total
population. As you notice this is evidence that mutational
robustness does not evolve in regime I, but why? And where do
these fluctuations come from exactly?***

**Response:** In Fig. 1, the number of host types was measured using the expression level of the target gene in the snapshot at a specific time interval, and thus, the values before the gene reached steady-state expression at c = 0, are also counted and other types besides the optimal phenotype are shown.

The previous figure could be misinterpreted as you rightly pointed out, and thus, we have replaced it by new figure using gene expression x after steady-state.　At c=0, there is no significant change in the population composition except for the appearance of mutants with low growth due to the absence of mutational robustness.

The population is dominated by hosts with the optimal phenotype at steady state, but the GRNs evolved at sigma = 0 is highly susceptible to genetic mutations. As studied in [17,23], the mutation robustness evolved following the developmental robustness. If the noise level is too small, the developmental robustness to noise does not evolve. Accordingly, the mutational robustness does not evolve either.

In this case, the GRN dynamics do not provide a globally stable attractor. The landscape evolved at σ < σc is rugged. This is already studied in [17,23] and reviewed in [26], and thus, it is not repeated here. Having said that, we have added a brief remark on page 13, referring to previous studies.

***- As mentioned above, in Fig. 4A, it would be nice to see the
behavior of V_ip' (variance due to on/off switches only), and in
particular, whether it increases as a function of sigma. If
V_ip' does not increase (or increases less than in random
networks) this would be evidence for developmental robustness.
In addition, we see that V_g decreases as a function of sigma
(mutational robustness). This might be either a correlated
response to increased developmental robustness, or (in case
developmental robustness is absent) and kind of "compensatory"
evolution (but this would mean that evolution of developmental
and mutational robustness can be decoupled, or at least that one
evolves easier than the other).***

**Response:** Thank you for your comment. We computed Vip’. As shown in Figure S9 the variance Vip’ of the non-target genes is decreased compared to the initial network, and the developmental robustness is increased. As for the non-target output genes, the developmental robustness of Vip' increased in correlation with Vg.

It should be noted that for sigma>sigma_c, even in the absence of parasites, the variance decreases while satisfying Vip>Vg and mutation robustness evolves as studied in [23].

On the other hand, Vip’ of the target genes increase compared to those in the initial network (random network).

Figure 3 shows that Vg of the target gene increases from the initial value regardless of the sigma. As we discuss in the next response, Vg decreases with sigma, but not more than the case with c = 0. Since both Vip' and Vg increase from their initial values, there exists some coupling between the two variances. Neither the evolution of "developmental robustness" nor "compensatory" evolution has occurred in the target gene.

We added some remark on this point at page 14.

***- In Fig. 5, as also mentioned above, it seems to me that V_ip'
does not increase beyond sigma = 0.02. In this case, why does
V_g decrease? Again, is this a correlated response to the
evolution of developmental robustness (which keeps V_ip
constant), or is there a different cause? For example, I have
been wondering whether V_g automatically decreases with sigma
(maybe mutations have less effect if expression levels
fluctuated all the time anyway?, see my question about random
networks above).***
**Response:** Fig. 6, which measures V_ip' against noise, shows that the transition due to noise-induced switching occurs when sigma $\geq$ sigma_c. V_ip' is the same level beyond sigma =0.02. As the reviewer commented, V_ip’ does not show a significant increase for larger sigma, as compared with V_ip, and V_g does not show an increase against sigma. This can be interpreted as follows: When there is no interaction (c = 0), Vg tends to decrease as sigma increases, as studied previously [23], and also shown in Supplement Fig. 8. This is because for larger noise strength, the acquisition of developmental robustness to noise is needed which leads also the increase in robustness to genetic mutations. The relationship between the two has been investigated in detail in a previous study [23] and also in review [26]. When c is nonzero, Vg is larger than the case for c = 0, but the decreases for the case with c = 0 with noise still remains, so that Vg shows slight decrease against sigma. We have added this discussion in the second paragraph on page 13.

***Some more question:
- In the presence of noise-induced variations, do expression patterns
remain for individual cells once they have settled down to a stable
state, or do they occasionally change? In other words, does
noise-induced variation manifest only in the differences between
isogenic individuals, or also in single individuals over time?***

**Response:** We have examined the time course of gene expression for each GRN after evolution, and found that once cells settle down to different fixed-point attractors, they do not show the switching through time. Depending on the developmental process from the initial values, the gene expression patterns may vary, which leads to a phenotypic variance in the population. We have added this information in the second paragraph on page 14.

***- In the absence of noise (sigma = 0), do the expression dynamics of***
***isogenic individuals always converge to the same pattern, or are
can there be differences cause by differences in the initial random
expression levels?*
Response:** If sigma=0, isogenic individuals converge to the same pattern. Initially, all gene expression levels are set smaller than the threshold \theta, and the time course of gene expression levels do not reach different phenotypes, without noise. We added a remark on this point in the second paragraph on page 7.

***Specific comments:
-----------------
- Equation (1): As mentioned above, for fixed $f$, this describes an
Ornstein-Uhlenbeck process (a random walk with a tendency to return
to the mean), in which $x_i$ fluctuates around $f + \epsilon$ with
variance $\sigma/(2\gamma)$.***

***- 93: Make it easier for the reader to understand that (i) there
are M (= 64) genes in total, (ii) the first $l_{out} = 8$ genes
are output genes affecting growth, (iii) of these the first $l_p =
3$ are target genes, and (iv) the last $l_{inp} = 8$ are input
genes.***

**Response:** We have added the description of this point in the first paragraph on page 4.

***- 96: Maybe the formula in this line is needlessly complicated
here, and it is enough to just say that $\eta$ describes white noise
which uncorrelated across genes. This is especially true if you
state that equation (1) describes an Ornstein-Uhlenbeck process (and
maybe mention that it is a Langevin equation, even though most
biologists will not know this term).***

**Response:** Following your remark, we removed the formula about $\eta$.

***- 94: k should be l_inp.***

**Response:** Thank you for pointing out the typographical errors. We have corrected the text following your suggestions.

***- 98: Does epsilon play any role at all for the results?***

**Response:** We have added epsilon to the simulation to prevent the expression x from touching zero.

***- 102: I = 1 means that input genes are always expressed.
(Indeed, the idea of external input seems unnecessary here; you
might just say that these genes are always/constitutively
expressed).***

**Response:** Following your remark, we have added the text in the first second on page 4.

***- 104: output genes have already been mentioned in line 94.***

**Response:** Following your remark, we removed this line.

***- 106: If I understand correctly, N = 300 is constant throughout,
as in the Moran model.***

**Response:** As the reviewer rightly pointed out, the value is constant throughout. We have added the description of this point in the first paragraph on page 4.

***- Equation (3) and (4): I suppose that the growth rate is evaluated
instantaneously at each moment and can change over time (so it should be $\hat \mu^{(l)}(t)$ in equation (5).***

**Response:** The growth rate can change over time. Following your remark, we corrected the equation.

***- 130-1: the part about parasite dynamics should be moved to the next
paragraph.***

**Response:** We have corrected the text following your suggestions.

***- 136: When there are 300 cells and one divides, the upper limit N
will always be exceeded, so it is more precise to say that whenever
a cell divides, another randomly selected dies.
- 140: What is s? Seconds? Maybe better to just say between 500 and
1000 time units.***

**Response:** We have corrected the text following your suggestions.

***- 141: When the gene-regulatory network reaches a steady state, but
there is phenotypic variation due to noise, do individual networks
settle into a stable state (which may vary between individuals), or
are genes occasionally switched on or off in response to
fluctuations in expression levels?***

**Response:** As we have already mentioned, both of these can occur throughout the evolution. Many individuals settle into a steady state in the last generation.

***- 149: alpha = 0.3 is not just an initial choice, but a choice that
remains valid throughout the simulation.***

**Response:** Thank you for pointing this out. We corrected the text.

***- Equation (6): c is the attack parameter from equation (4). Here it
should probably be replaced by 1/T_p.***

**Response:** Thank you for pointing this out. We replaced it by $1/T_p$ in equation (6).

***- 178: As mentioned above, V_ip has two components: One to the
fluctuations in eta, and one due to noise-induced on/off
switches.***

**Response:** Following your remark, we added the text of V_ip

***- 185: V_g is not the variance due to genetic differences, but the
variance induced by mutations. In particular, unlike V_{ip}, V_g
is not expressed within a generation, but only between generations.
It is really important to make this clear, especially since readers with a
background in quantitative genetics will tend to assume that V_g is
the genetic variance (i.e., the variance in $\bar x$), which is not
the case.***

**Response:** Thank you for pointing this out. We have replaced the misleading words “genetic differences” with variance due to genetic mutations.

***- Table 1: Is the mutation rate m = 0.0002 (as in the table) or 0.03
(as in line 143)?***

**Response:** Thank you for pointing out the typographical error. We have revised m as 0.03.

***- 200: From which sample? The one for t = 0?***

**Response:** Following your remark, we corrected the text.

***- 241: Again, V_g is not the genotypic variance, but the variance
induced by new mutations.***

**Response:** Following your remark, we corrected the text.

***- 247: Initial values of V_g are high because random networks are not
canalized and highly sensitive to mutation.***

**Response:** Following your remark, we corrected the text.

***- 266-7: V_ip is higher, not lower, under large sigma for small c in Fig. 4
(regime 2).***

**Response:** Following your remark, we corrected the text.

***- 268: Where can I see that V_ip and V_g both decrease (the purple
lines in Fig. 2?)***

**Response:** Following your remark, we added the new figure of V_ip and V_g for c=0 in Fig. S8.

***- 286: c = 4?***

**Response:** Following your remark, we corrected the text.

***- 152: Unlike host dynamics, which are stochastic and
individual-based, parasite dynamics are modelled via a deterministic
model of genotype frequencies.
- 177-180: These two sentences seem redundant, and they disrupt the
connection between "both from noise and from network mutations"
(177) and "We define two phenotypic variances ..." (180).
- 235: the number of coexisting host genotypes approaches the maximal
number 2^{lp} = 8, corresponding to the total number of parasite
genotypes.
- 259: are not
- 312: GRNs that became extinct due to parasite infections are excluded
- 317: and robustness increased
- 320: Fig. S6
- 324: whether high variability in phenotype can evolve in response
to...
- 327: once the host had adapted
- 331: before increase phenotypic variation evolves
- 332: Thus, notwithstanding ..., phenotypic fluctuations can evolve
in response to parasite infection
- 338: A comparison of Fig. 6[i] and [ii] shows the final variances
are nearly identical. This means that the evolution variances in
both target and output genes is independent of the initial
conditions.
- 358: the expression of target genes is variable***

**Response:** Thank you for pointing out these typographical errors. We have corrected all of them.
