# Supplementary material for: Evolution of phenotypic fluctuation under host-parasite interactions
Source: PLoS Comput Biol. 2021 Nov 9;17(11):e1008694. doi: 10.1371/journal.pcbi.1008694 (PMC8604345; doi:10.1371/journal.pcbi.1008694)
Supplement: Attachment — Submitted filename: responce to reviewers.docx [file pcbi.1008694.s005.docx]

We thank the reviewer for the time and effort put in towards the review of this manuscript. We have incorporated several changes based on the comments of the reviewers. The detailed responses to the reviewer comments are given below.

***In this revision, the authors have answered most of my questions. In***

***particular, the new Fig. is a real improvement, which shows that the***

***variance due to noise-induced on/off switches does not increase for***

***noise levels beyond sigma = 0.02. In would be good to discuss why this***

***is so. One potential explanation is that the attained level of***

***noise-induced phenotypic variation, in conjunction with genetic***

***variation, is enough to produce the maximal phenotypic variation with***

***8 coexisting target-gene expression patterns.***

Thank you for your comment. Based on your remark, we added the description of the reason why noise-induced on/off switches does not increase for

noise levels beyond sigma = 0.02 in the second paragraph on page 13.

**Editorial comments:
------------------**

***- 345-351: I don't understand what you are trying to say here.***

We thank the reviewer for pointing out our misleading descriptions and insufficient explanations. We added the correct explanation in the second paragraph on page 13.

***- Abstract, first "blue" sentences: two typos in plasticity***

Thank you for pointing out these typographical errors. We have corrected all of them.

***- Abstract, line 9: where one of the most important...
 - Abstract, second "blue" part: By using a dynamic model of gene
expression for the host ... evolution of the genotype-phenotype map
and of phenotypic variances
 - 8: Maybe: In this context, phenotypes are shaped by dynamic
processes involving several genetically determined variables, as
well as external and internal noise. Hence phenotypes can
vary not only as a consequence of genetic variation, but even
between isogenic individuals [6–10]. The sum of both sources of
phenotypic variation defines the genotype–phenotype mapping [14–16].
 - 31: remain
 - 46: latter's
 - 81: I still think it would be useful for readers to say that, for
constant f, equation 1 defines an Ornstein-Uhlenbeck process, and to
give the equilibrium variance of sigma^2/(2gamma).
 - 92: total gene number***

***- 110: constant at N = 300 throughout all simulations
 - 125: Let m be the parasite type
 - 142: after around 200 time units
 - 145: value
 - 145: one of the others
 - 151: alpha = 0.3, and alpha remains valid...
 - 151: of this choice
 - 153: Unlike host phenotype (gene expression levels), which is
partially stochastic, the parasites' phenotype is determined uniquely by
its genotype. Hence parasites of a given genotype share a common
phenotype and parasite population dynamics can be modelled by
deterministic equations (instead of individual-based simulations).
 - 192: the variance ... within a population of initial genotype i in
which each individual receives one random mutation.
- 199: Note that developmental noise (the sigma eta term in equation
1) has no significant effect on host fitness once gene expression
dynamics have reached a stable state with all x_i close to 0 or 1.
This is because host growth depends only on mean gene expression
levels and parasite attack depends on rounded values (whether x_i is
< or > 0.5). However, noise is important, because when amplified in
the GRN, it may cause the initial dynamics to converge to a
different stable state (i.e., noise-induced switches in gene
expression). Regaring phenotypic variances, we discuss two types of
robustness: mutational robustness and developmental robustness.
Mutational robustness describes the "ability" the ability of a GNR
to prevent changes in gene-expression patterns due to mutations in
the network structure, and developmental robustness describes the
ability to prevent changes in expression patterns due to white-noise
fluctuations in gene expression level during the initial network
dynamics.
 - 209: We started simulations with N host cells of an initial
gene-regulatory network created by creating random paths with
probability alpha = 0.3.
 - 210: What are the 50 samples? 50 individuals from a population, or
50 replicated populations?
 - 211: The initial condition for each cell is given by a state in
which only the input genes are expresses: all other genes are
initialized with expression levels drawn uniformly between 0 and
0.05. This same initial condition was also applied to the daughter
cells after each cell division.
 - 214: that went extinct
 - 216: add "in the absence of noise (sigma = 0)" at the end of the
sentence
 - 229: distribution of parasite genotypes
 - 231: Note that in the absence of noise...
 - 233: Next, we computed
 - 233: expression levels
 - 236: Independent of the level of developmental noise, the Figure
shows a marked increase...
 - 238: This sentence is redundant
 - 255: These correspond to two strategies
- Fig. 3, new sentence: Note that the plot starts with the first
rather than te 0th generation, and that host types that went extinct
during the simulation have been exluded. This explains the different
initial variance levels between parameter combinations.
 - 261: after evolution of the system reaches a stable state
 - 263: These results are independent of the choice of the initial
network
 - 287: to both noise and mutation
 - 294: whereas V_ip remains small
 - 299: Maybe: For moderate c > c_t, the genotypes with the fittest
type 111 coexists with genotypes having one target gene switched off
..., whereas for c = 4, even more host types appear, leading to
coexistnence of all eight target gene expression patterns.
 - 307: The sentence starting with "In addition" seems redundant with
respect to the previous one.
 - 309: Variances in expression levels are highest for target genes
 - 320: Note that, in Fig. 5, V_g decreases slightly with sigma once
sigma exceeds the critical value sigma_c. This reflects previous
results for c = 0 (i.e., in the absence of parasites), which showed
that mutational robustness decreases as a correlated response to the
evolution of developmental robustness against increasing noise
levels (Fig. S8).
 - 329: As mentioned above, once expression levels of host target genes
have settled down close to one of the fixed points x = 0 or 1,
continued variation due to the white-noise term in Fig. 1 has no
effect on the interaction with the parasite. The noise is important,
however, for the initial network dynamics that determine which of
these fixed points is approached in the first place. In order to
separate the contributions of noise-induced on/off-switches from
those of the noise itself, we define a variance V_ip' which only
reflects the former. More precisely, V_ip'(i) is the variance
of the on/off variable z_i = ..., computed in the same manner as
V_ip(i).
 - 336: After the first sentence, first discuss Fig. 6: "The key
difference to Fig. 5 is that for sigma > 0.02, V_ip' remains
essentially constant for both target and output genes, suggesting
that the variance due to noise-induced switches cannot be increased
arbitrarily, and that the continued increase of V_ip seen in Fig. 5
is merely a reflection of the increase in white noise."
- Then go on to discuss Fig. S9: E.g., "Evolutionary time
courses for both V_ip and V_ip' are shown in Fig. S9. For target
genes, both measures increase from their initial value and then stay
at a high level, with no evidence for evolution of developmental
robustness. For non-target output genes, in contrast, V_ip and V_ip'
decrease over time relative to the values in the initial random
networks. This shows that ..."
 - 352-355: Thanks for adding this, but it be better placed earlier in
the paper, for example in the model description, after line 151.
- in the 0th generation?
- 368: closer than what?
 - 370: remove "the" before developmental robustness
 - 379: after developmental robustness has evolved
 - 381: and let the system evolve
 - Caption Fig. 7: It seems that there is a problem with the sentence
right after the blue insert.
 - 418: within a regulatory network
 - 421: the phenotypic variance of the host increases as strains evolve
adaptation to decrease attack by specific parasites.
 - 427: which enhances phenotypic variation and helps...
 - 428: The new sentence seems redundant.
 - 430: both variances***

We thank the reviewer for pointing out our misleading descriptions and insufficient explanations. Based on your remark, we have corrected all of them. We believe that the present revision throughout the manuscript will meet all of your requests. We would be grateful if you consider the publication of the present version.
